# Prenatal Diagnosis of Congenital Syphilis Using Two- and Three-Dimensional Ultrasonography: Case Report

**DOI:** 10.1155/2012/478436

**Published:** 2012-08-21

**Authors:** Edward Araujo Júnior, Eduardo Felix Martins Santana, Liliam Cristine Rolo, Luciano Marcondes Machado Nardozza, Antonio Fernandes Moron

**Affiliations:** Department of Obstetrics, Federal University of São Paulo (UNIFESP), Rua Carlos Weber, 956 apto. 113 Visage, Vila Leopoldina, São Paulo 05303-000, SP, Brazil

## Abstract

The numbers of syphilis cases have been increasing considerably, especially in eastern europe, thereby contributing towards greater chances of cases of congenital syphilis. Some of the complications of congenital syphilis can be detected on two-dimensional ultrasonography (2DUS), and these are generally manifested in the second trimester of pregnancy. The commonest ultrasonographic signs are hepatosplenomegaly, placentomegaly, and fetal growth restriction, while lower-frequency occurrences include intrahepatic calcifications, ascites, fetal hydrops, and even fetal death. Three-dimensional ultrasonography (3DUS) is a relatively new imaging technique that is adjuvant to 2DUS and enables detailed assessment of the fetal surface anatomy. We present a case of a 21-year-old primigravida with a diagnosis of congenital syphilis, with obstetric 2DUS findings of hepatosplenomegaly, ascites, pericardial effusion and hyperechogenicity of the cerebral parenchyma. 3DUS in rendering mode allowed clear assessment of the fetal limbs, especially the feet, which appeared twisted and lacked some toes. It allowed the parents to understand the pathological condition better and improved prenatal management and neonatal followup. 3DUS can be used routinely for assessing fetal malformations resulting from congenital infections.

## 1. Introduction

The advent of penicillin gave rise to a notable decline in the incidence of sexually transmitted diseases. However, in developing countries, the numbers of syphilis cases have been increasing considerably, thereby contributing towards greater chances of cases of congenital syphilis [1–3]. Some of the complications of congenital syphilis can be detected using two-dimensional ultrasonography (2DUS), and they are generally manifested in the 24th week of gestation [4]. The commonest ultrasonographic signs are hepatosplenomegaly, placentomegaly, and fetal growth restriction, while lower-frequency occurrences include intrahepatic calcifications, ascites, fetal hydrops, and even fetal death [4–6]. Three-dimensional ultrasonography (3DUS) is a relatively new imaging technique that has been used to identify a variety of fetal abnormalities prenatally [7, 8]. It has the advantage of producing reconstructed images in rendering mode, which allows the parents to understand the fetal malformations better and thus facilitates genetic counseling [7, 8]. However, no descriptions of the use of this new method in prenatally diagnosing congenital syphilis have yet been published. 

We present a case of congenital syphilis that was diagnosed in the third trimester of pregnancy, in which we cover the main findings from 2DUS and present the 3D imaging. We focus on the importance of 3DUS in diagnosing the fetal malformations resulting from congenital syphilis, which was of great importance in providing counseling for the parents, and in the prenatal followup and obstetric management. 

## 2. Case Presentation

**Figure 1:**
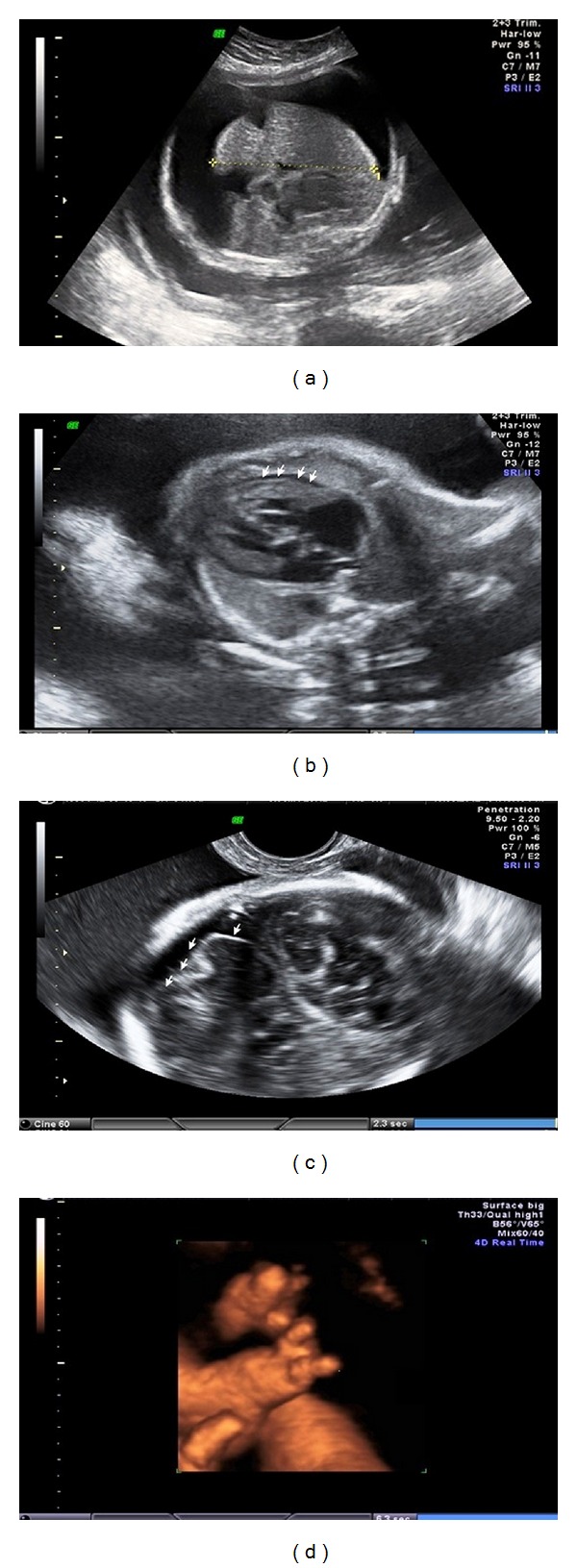
(a) Axial plane of the fetal abdomen on 2DUS, showing ascites and hepatomegaly. (b) Axial plane of the fetal thorax on 2DUS at the level of the four chambers of the heart, showing pericardial effusion (white arrows). (c) Sagittal plane of the fetal cranium on 2DUS, showing hyperechogenicity of the cerebral parenchyma. (d) Three-dimensional image in rendering mode, showing twisting and absence of some toes.

The patient was a 21-year-old primigravida who was referred to the high-risk prenatal service of the Department of Obstetrics of the Federal University of São Paulo (UNIFESP) because of a finding of fetal ascites on morphological ultrasonography performed in the second trimester of pregnancy. In a routine prenatal serological test performed in a public healthcare clinic, VDRL 1/32 was detected in the 7th week of pregnancy. However, for socioeconomic reasons, the treatment for syphilis was only started in the 25th week of pregnancy, using benzathine penicillin (total dose of 7,200,000 UI). VDRL was 1/4 after this treatment. Morphological ultrasonography performed at another service in the second trimester detected a separate finding of fetal ascites. In the 31st week of pregnancy, when the woman was referred to UNIFESP's high-risk prenatal service, ultrasonography was performed again and the following fetal abnormalities were detected: voluminous ascites, splenomegaly, pericardial effusion, twisting of the toes, and hyperechogenicity of the cerebral parenchyma (Figures 1(a), 1(b), and 1(c)). In order to better assess the surface malformations, 3DUS was performed using the Voluson E8 apparatus (General Electric Medical Systems, Zipf, Austria), equipped with a volumetric convex transducer (RAB 4–8 L). The 3D imaging in rendering mode enabled clear assessment of the fetal limbs, especially the feet, which appeared twisted and lacked of some toes (Figure 1(d)). Three-dimensional imaging was of great importance in enabling the parents to understand the pathological condition better, and this allowed us to provide better counseling for prenatal care, obstetric management, and neonatal followup.

New serological tests for syphilis were performed in the 32nd week. VDRL was 1/64 and the specific treponemal test (ELISA-TPMA) was positive. The patient was then hospitalized for new treatment with benzathine penicillin (7,200,000 UI) and monitoring of fetal vitality. Cordocentesis performed in the 33rd week confirmed the diagnosis of fetal syphilis. Because of the many structural abnormalities presented by the fetus, it was decided to perform cesarean delivery at term. The newborn presented adequate vitality, weight of 3,095 g, and Apgar of 9/10 and was admitted to the neonatal intensive care unit (ICU). In the ICU, the newborn underwent paracentesis and spinal tap, from which the specific treponemal test and VDRL were positive. Cytological tests on the fetal cerebrospinal fluid showed elevated cellularity. The newborn was kept in hospital for 14 days, receiving crystalline penicillin, and was then discharged without presenting any intercurrences.

## 3. Discussion

Because of the epidemiological importance of syphilis, the increasing incidence observed in some countries is a matter for concern, especially when associated with the gestational and neonatal complications that congenital syphilis may gave rise to [1, 2]. Since this disease may have an impact on populations, the World Health Organization has declared that investigation of *Treponema pallidum* should be mandatory for all pregnant women, among other serological tests that are necessary [1, 2]. Syphilis can be diagnosed using nonspecific tests (VDRL) and specific tests (FTA-ABS or ELISA-TPMA). In suspected cases of congenital syphilis, the diagnosis can be confirmed by the presence of *Treponema* in the amniotic fluid (amniocentesis) and in the fetal blood (cordocentesis), through dark-field examination and treponemal tests, respectively [9, 10]. If congenital syphilis is suspected or diagnosed prenatally, neonatal confirmation of the diagnosis becomes necessary (VDRL in the fluid, IgM FTS-ABS in serum, dark-field examination of skin lesions, secretions and fluids, and radiological examination of long-bone lesions) [4].

In addition to laboratory diagnostic tests, 2DUS performed at the same stage of morphological screening in the second trimester may detect the main abnormalities resulting from congenital syphilis that have been described in the literature: hepatosplenomegaly, placentomegaly, fetal growth restriction, ascites, intrahepatic calcification, and intracerebral calcifications [3–6]. 

From our case, we present here a prenatal image of congenital syphilis produced using 3DUS, which has not been shown in the literature previously. 3DUS has been used over the last few years as a prenatal diagnostic technique complementary to 2DUS and has enabled better assessment of fetal surface abnormalities [11]. In our specific case, 3DUS clearly demonstrated the twisting and absence of some toes. This made it easier for the parents to understand the abnormalities, and it helped the obstetric team to provide better prenatal care, and management, and helped in the prenatal followup.

The treatment for congenital syphilis is based on antibiotic therapy, and the preferred drug is benzathine penicillin in most cases. Crystalline penicillin is used in cases that are more advanced [12]. However, there is a chance of treatment failure, even when adequately instituted in the third trimester of pregnancy. For this reason, for there to be a real reduction in the rates of congenital syphilis, rigorous prenatal followup of pregnant women with syphilis is necessary, with serial control serological tests and ultrasound scans. An active approach is recommended, in order to identify and treat newborns that have been exposed to this.

In summary, we presented a case of congenital syphilis that was diagnosed prenatally by means of 2DUS and 3DUS. We believe that 3DUS can be used routinely in cases of prenatal congenital infections (and not just in cases of syphilis), so as to enable the parents to better understand the abnormalities presented by the fetus, and to assist the obstetric team in terms of prenatal followup, delivery, and neonatal followup.
